# Synaptic plasticity and mental health: methods, challenges and opportunities

**DOI:** 10.1038/s41386-022-01370-w

**Published:** 2022-07-09

**Authors:** Lawrence G. Appelbaum, Mohammad Ali Shenasa, Louise Stolz, Zafiris Daskalakis

**Affiliations:** Department of Psychiatry, University of California, San Diego, La Jolla, CA USA

**Keywords:** Depression, Schizophrenia, Adult neurogenesis, Neurophysiology

## Abstract

Activity-dependent synaptic plasticity is a ubiquitous property of the nervous system that allows neurons to communicate and change their connections as a function of past experiences. Through reweighting of synaptic strengths, the nervous system can remodel itself, giving rise to durable memories that create the biological basis for mental function. In healthy individuals, synaptic plasticity undergoes characteristic developmental and aging trajectories. Dysfunctional plasticity, in turn, underlies a wide spectrum of neuropsychiatric disorders including depression, schizophrenia, addiction, and posttraumatic stress disorder. From a mechanistic standpoint, synaptic plasticity spans the gamut of spatial and temporal scales, from microseconds to the lifespan, from microns to the entire nervous system. With the numbers and strengths of synapses changing on such wide scales, there is an important need to develop measurement techniques with complimentary sensitivities and a growing number of approaches are now being harnessed for this purpose. Through hemodynamic measures, structural and tracer imaging, and noninvasive neuromodulation, it is possible to image structural and functional changes that underlie synaptic plasticity and associated behavioral learning. Here we review the mechanisms of neural plasticity and the historical and future trends in techniques that allow imaging of synaptic changes that accompany psychiatric disorders, highlighting emerging therapeutics and the challenges and opportunities accompanying this burgeoning area of study.

## Overview

Neuroplasticity has a profound impact on the manifestation and progression of clinical symptoms in many psychiatric illnesses. Understanding the multiscale role that plasticity has on neuropathology is critical to creating improved, targeted therapies. Through experiments that directly manipulate plasticity to create testable, causal changes over time, and through cross-sectional, retrospective, and postmortem analysis that reveal accumulated differences in plasticity that arise naturally, it is possible to obtain valuable information about the mechanism-of-action underlying healthy and disordered synaptic plasticity. While there has been considerable progress toward understanding these mechanisms, the impact of plasticity on behavior and the best approaches to induce neuroplastic change for therapeutic gains still remain open and important questions. This review seeks to provide a contemporary overview of the science elucidating the mechanisms of plasticity, the current state-of-the-art in methods used to image plasticity and promote therapeutic gains, and to provide suggestions for profitable future directions.

## Mechanisms of neural plasticity

Neural plasticity refers to the possibility of altering the strength of connections within the nervous system through experience or injury. Plasticity can act to reorganize either the structure or the function of neurons and is necessary not only for neural networks to acquire new capabilities, but also for them to remain robust and stable over time. A key element of plasticity revolves around the temporal coincidence of activity. So-called, spike-timing-dependent plasticity (STDP) is a Hebbian learning rule in which the modification of synaptic strengths depends on the relative timing of action potentials [1]. Within monosynaptic pairs of neurons, it has been shown that if an input spike from the presynaptic neuron occurs immediately before the postsynaptic neuron’s output spike, then that input becomes stronger, creating long-term potentiation (LTP). If the input spike occurs immediately after an output spike, however, that input is made weaker, creating long-term depression [2, 3] (LTD) (While STDP generally involves molecular alterations at the synapse they rely on wide variety of distinct mechanisms that can differ in different brain regions (e.g., LTP induced during learning is different in the hippocampus and amygdala), among different neurons in the same brain region (e.g., endocannabinoid and non-endocannabinoid LTD in projections from the striatum to the basal ganglia), or within the same types of neurons (e.g., hippocampal CA3 pyramidal neurons that converge from different afferent inputs)). This critical window of timing-dependency spans tens of milliseconds and has profound functional implications on brain function, creating a means for activity-dependent bidirectional modification of synaptic strength, and ultimately forming the physiological basis for learning and memory. The mechanism underlying STDP has been attributed to two different glutamate receptors that are commonly co-expressed, the α-amino-3-hydroxy-5-methyl-4-isoxazolepropionic acid (AMPA) receptor and the N-methyl-D-aspartate (NMDA) receptor [4]. The NMDA is a glutamate receptor cation channel that is also widely referred to as a “coincidence detector”. Within this channel, coincidence is detected by the simultaneous presence of both membrane depolarization that vacates a channel-blocking magnesium ion, and the binding of its natural ligand, glutamate [4, 5]. The preexisting membrane depolarization of NMDA is in turn mediated through the coactivation of AMPA activation [6] to create reciprocal cellular mechanisms that enable long-term synaptic changes.

Beyond spike-timing-dependent plasticity that occurs rapidly at the synapses, other slower homeostatic processes occur over hours, days, or weeks to modify ion channel density, neurotransmitter release, or postsynaptic receptor sensitivity [7]. These processes are triggered in response to rapid activity-dependent changes and constitute a negative feedback loop, decreasing connectivity in response to high neuronal activity but increasing connectivity when activity drops [8]. Yet, another plasticity-based moderator of neuronal network communication is the growth of myelin, a multilayered membrane produced by oligodendrocytes that surrounds axons to increase the speed by which electrical signals propagate through the nervous system. While widely associated with critical developmental periods, there is also considerable evidence that activity-dependent myelination continues into adulthood [9] and can be negatively impacted by psychiatric (e.g., schizophrenia and bipolar disorder) and neurodegenerative (e.g., Alzheimer’s) diseases [10]. Though the relative contribution of growth factors, extracellular signaling molecules, and neuronal activity of myelination is still currently unknown [11], emerging methodologies discussed below are beginning to offer new tools to answer these questions.

Together, these Hebbian and homeostatic processes, and their interaction with neuronal oscillations and neurotransmitter receptor signaling allow for the essential ability of the nervous system to modify itself, functionally and structurally. This, in turn, leads to changes in neural architecture that gives rise to mental function, is sculpted through development, and is durable in response to the changing environment, aging, or pathological insult.

## Disordered synaptic plasticity that contributes to mental health disorders

Synaptic plasticity is intrinsic to the development and function of the brain, conferring environmental adaptability, learning, and overall well-being. However, exposure to environmental factors such as stress, psychological trauma, substance use, and other sociocultural influences can have profound influences on brain plasticity leading to a host of psychiatric and mental health disorders. Unlike the brain pathology that results from trauma or stroke, mental health disorders and addictive disorders do not result from specific localizable lesions in the brain, but rather are characterized by distributed pathology, particularly in limbic, prefrontal, and frontostriatal areas that support perception, cognition, motivation, and the regulation of emotion. Also, unlike neurological trauma, the pathogenesis of mental health and addictive disorders are typically associated with polygenic risk factors that are strongly influenced by neurodevelopment and environmental experiences [12]. Moreover, the clinical trajectory of mental health illness and the underlying brain dysregulation tends to be chronic, recurring, and episodic, with slow recovery and high rates of relapse [13, 14]. As such, neuroplasticity has both a large influence on the genesis of mental health disorders and serves as a possible mechanism that can be targeted to achieve therapeutic gains.

Past research has begun to elucidate the specific role that synaptic plasticity mechanisms play in psychiatric disease expression [15, 16]. For example, both major depressive disorder (MDD) and posttraumatic stress disorder (PTSD), two disorders that demonstrate a high level of comorbidity and shared neuropathology, have routinely demonstrated synaptic loss in dorsolateral prefrontal cortex (DLPFC) circuits that underlie affective and cognitive processes [17]. In support of this, evidence from cross-sectional brain imaging studies consistently show lower brain volume in the DLPFC, anterior cingulate cortex, and the hippocampus in both MDD [18, 19] and PTSD patients [20], while analysis of functional connectivity (FC) between PFC and limbic areas is frequently shown to be reduced in both MDD [21] and PTSD [22]. Similar approaches have demonstrated prominent disturbances in DLPFC circuitry in schizophrenia [23] with emerging genomic research pointing to genes that regulate Brain-derived neurotrophic factor (BDNF) and the NMDA receptor, as specific mechanisms that might contribute to these abnormal patterns of cortical connectivity observed in schizophrenia [24].

Addiction, like other mental health disorders is rooted in neuropathology that interacts with environmental experiences. Through repeated pharmacological insult stemming from drug and alcohol use, changes occur to the brain circuits that regulate how a person interprets and responds to motivationally relevant stimuli. This in turn leads to systemic alteration of in neurotransmitter uptake that affects reward circuitry in the brain. In particular, repeated substance use causes increased release of dopamine from cells in the ventral tegmental area into the prefrontal cortex, amygdala, and striatum [25]. This association between increased dopamine transmission and reward produces long-term plastic changes that increase the biological motivation for the craved substance while also driving up tolerance to the pharmacological agent, increased psychological dependence, and withdrawal symptoms when the addicted substance is absent [26].

Collectively, these and other findings characterizing the dysregulation in synaptic plasticity and associated changes in brain dynamics underlying psychiatric illness lay a framework for how interventions may affect synaptic function and impact plasticity. In the following two sections we review methods used to image and modulate synaptic plasticity that contributes to mental health disorders and refer the reader to other articles in this special issue for deeper treatment of the role of disordered and maladaptive plasticity in specific psychiatric disorders.

## Imaging synaptic plasticity in the human brain

Modern neuroscience has created several techniques for imaging synaptic plasticity in the human brain. This discussion begins with a description of in vitro studies to establish a working model for measuring changes in synaptic strength, followed by sections on magnetic resonance imaging (MRI) and positron emission tomography (PET), two noninvasive approaches that have shown promise for imaging synaptic plasticity.

### Synaptic plasticity as seen in vitro studies

In vitro studies of neurons within the human neocortex demonstrate that high-frequency stimulation potentiates neurons, while low-frequency stimulation de-potentiates synaptic strength [27]. This heuristic is bidirectional, meaning each form of plasticity may also dynamically reverse the other in response to stimulation. For instance, high-frequency stimulation is able to potentiate synapses having undergone LTD, and vice versa. The primary event that actualizes LTP and LTD is the respective insertion and removal of AMPA receptor [4]. These findings, measured by intracellular excitatory postsynaptic potentials and extracellular field potentials, provide a model of how LTP and LTD manifest by demonstrating increased or decreased electrophysiological responses, respectively [28]. The in vitro models for induction of synaptic plasticity have been remarkably consistent with mechanisms seen in vivo studies.

### MRI modalities and their applications to imaging brain plasticity

The plasticity of large-scale networks can be effectively studied through connectivity and tractography analysis of MRI data. This large and growing body of literature has demonstrated the characteristics of healthy function and disease states on the brain’s functional architecture [29] and differences in tract integrity are a hallmark of many psychiatric disorders [30, 31].

Various MRI modalities that can image synaptic plasticity include structural, diffusion, and functional MRI. Structural MRI allows measurement of the volume and thickness of brain structures. For example, gray matter increases, as measured through voxel-based morphometry (VBM), have been shown to be reflective of neuroplasticity in adults after task learning [32]. In particular, cortical gray matter has been noted to increase during learning over weeks to months, with such changes believed to be secondary to synaptogenesis and dendritic arborization [33]. Diffusion MRI allows for analysis of white matter microstructure and tracts. Structural white matter changes have been visualized using diffusion tensor imaging during skill learning, which are believed to represent changes in conduction velocity and neural synchrony [33]. Finally, functional MRI assesses the strength and localization of brain function through task-related blood oxygen-level dependent (BOLD) signal, given that neuronal activity leads to local oxygen consumption, and in turn, increased compensatory blood flow.

There is considerable evidence demonstrating that changes in FC are reflective of experience-dependent plasticity resulting from synchronous activation between brain regions. Spontaneous, temporally correlated BOLD fMRI measurements across brain regions may be used to determine intrinsic FC or resting-state functional connectivity when collected explicitly at rest. Through dynamic causal modeling, effective connectivity may be used to assess directionality of connections among brain networks [29]. Combining functional and structural imaging methods may be used in conjunction to monitor alterations in plasticity. These methods have also demonstrated sensitivity to detecting BDNF and NMDA mediated alterations in plasticity, opening an avenue for testing pharmaceuticals aimed at altering plasticity [33].

To continue to image synaptic plasticity in the brain and relate changes to disease and learning, future endeavors should promote the use of rigorous study designs with randomization and active control, transparency of analytical methods, and measurement of physiological changes that may perturb MRI readings. This includes measures such as cardiac output and age-related developmental changes in MRI metrics that may inform differences in findings between children and adults. For instance, individuals with a genetic predisposition for Alzheimer’s disease have been shown to display a distinct neuroanatomical signature of a thinner entorhinal cortex, as seen as early as childhood [34]. Another example demonstrated that, preadolescent brains have an increase in cortical thickness as opposed to the thinning seen in young adulthood [32]. This is especially salient considering developmental plasticity may overlap with experience-dependent plasticity, reinforcing the need for well-matched control subjects to account for physiological age-related discrepancies in MRI metrics.

### PET imaging of synaptic density

Synaptic density can be measured through PET imaging of synaptic vesicle glycoprotein 2A (SV2A). SV2A is a presynaptic protein robustly expressed in glutamatergic and GABAergic neurons of the central nervous system. Previously, imaging of SV2A has been used to illustrate synapse loss in the hippocampus of patients with Alzheimer’s disease and the substantia nigra of patients with Parkinson’s disease, when compared to healthy controls [35, 36]. This approach has also been used to demonstrate decreased synaptic density in patients with schizophrenia, and synaptic loss in patients with temporal lobe epilepsy, showing its diverse applicability across a variety of pathologies related to alterations in synapses [36, 37]. In a recent study, SV2A-measured synaptic density was used to assess the antidepressant effects of ketamine in healthy controls and patients with MDD and PTSD. Interestingly, despite significant symptom reduction, there was no associated change in SV2A density however, post-hoc analyses suggested that ketamine’s therapeutic effect may lie in restoring synapses in patients with lower baseline SV2A levels [38].

The main challenges in clinical use of this imaging modality are due to the lack of validation studies. [^11^C]UCB-J is one of the most commonly used radioligands measuring SV2A, however, due to the short half-life of ^11^C, its availability to research sites is limited. [^18^F]UCB-H however, does have a longer half-life but a lower specific binding signal for SV2A. Therefore, there is a need for improved radioligands for future studies [39]. Furthermore, quantifying age-related synaptic density changes over time is also a key step in moving forward to have well-delineated controls [35]. Overall, SV2A PET imaging is a reliable technique for characterizing gross pathology and lends itself to exciting opportunities that may revolutionize diagnosis, staging, prognosis, and response to treatment in various neuropsychiatric disorders [40].

## Therapeutics and their relationship to plasticity

Given the profound influence of synaptic plasticity in psychiatric disorders, there is a central role for therapies that target dysregulated mechanisms to restore and maintain synaptic homeostasis. The following sections provide a mechanistic overview of synaptic plasticity that can be used to guide therapeutic development, followed by discussion of evidence for modulation of synaptic plasticity within pharmacologic, noninvasive brain stimulation (NIBS), and behavioral treatments. We refer the reader to other articles in this special issue for elaborated discussion of the role of plasticity in therapeutic effects for specific disorders such as depression and Schizophrenia.

### Mechanistic roles behind changes in neural plasticity and clinical improvement

As a framework for plastic changes, mechanisms underlying increased or decreased connectivity can be stratified into two levels: molecular and cellular levels. Molecular mechanisms are implemented via signaling pathways, resulting in gene transcription and protein synthesis of processes essential for plasticity. The resulting proteins lead to cellular changes, which can be further categorized into structural or functional plasticity. The former includes structural changes to neurons, such as neurogenesis and dendritogenesis, whereas the latter refers to changes in neuronal function through long-term potentiation or depotentiation, and in turn synaptogenesis [41]. BDNF is involved in all levels of neuroplasticity including neurogenesis and dendritic growth and is thought to be the primary modulator of synaptic plasticity [42]. It also serves as a useful biomarker of plasticity in human clinical trials.

An increase in BDNF is a ubiquitous consequence of many established and effective psychiatric treatments. For example, selective serotonin reuptake inhibitors, ketamine, and lithium have all been shown to increase BDNF levels [41, 43, 44]. Further, NIBS techniques that use exogenous electrical and magnetic pulses to the cortex have demonstrated excitability changes, or alterations in plasticity, that have been shown to be NMDA dependent, and mediated by BDNF secretion [33]. In fact, even exercise induced plasticity seen through learning and memory formation is dependent on hippocampal BDNF signaling, mediated by lactate produced via muscle metabolism and transported through the blood brain barrier [45], illustrating the ubiquitous role of this growth factor in nearly all facets of neuroplasticity.

Psychedelic substances including ayahuasca, N-dimethyltryptamine, psilocybin, and lysergic acid diethylamide are gaining increasing interest as clinical treatments that produce their therapeutic effects through induction of synaptic plasticity [40]. A systematic review by de Vos et al. includes 20 pre-clinical studies wherein a single administration of a serotonergic (5-HT2A) agonist psychedelic was found to induce rapid changes in plasticity on cellular and molecular levels, including gene expression of plasticity-related proteins such as BDNF. Mechanistically, these changes are proposed to result from activation of the 5-HT2A receptor, primarily in cortical glutamatergic pyramidal cells, leading to intracellular signaling pathways that ultimately facilitate postsynaptic insertion of AMPAR [46, 47]. Accordingly, these postsynaptic changes are considered functional, synaptogenetic changes, wherein the increased AMPAR density yields a strengthened synaptic connection and increased cortical BDNF release which further facilitates induction of plasticity [41, 46, 48]. Of note, the BDNF levels sampled in the studies reviewed by de Vos et al. were primarily peripheral, or indirect, levels. This is opposed to cerebrospinal fluid (CSF) BDNF levels, which are more invasive yet more precise given they more directly represent the brain chemistry. Among the studies that examined CSF and plasma BDNF levels, two studies have found a positive correlation between CSF and plasma BDNF levels [49, 50], and others indicated a positive relation with clinical response [51]. While a correlation between BDNF levels and clinical improvement is not a meaningful indicator of causality between the two, it may be the case that plasma BDNF is indeed an adequately accurate marker to be used, especially considering the alternative invasive nature of CSF monitoring.

In summary, the final common pathway of plasticity driven changes in response to many effective and newly emerging therapies appears to involve BDNF. Future studies should delineate differences in sensitivity and specificity of serum and CSF BDNF levels, as well as the rigor with which they predict clinical response to plasticity driven therapies. Additionally, correlating these levels with clinical imaging studies, including MRI and PET, is likely to provide the most thorough assessment of neuroplasticity driven changes in response to therapeutics.

### Neuromodulation and neurocircuitry-based treatments

Neuromodulation and circuit-based treatments, using both electrical and magnetic stimulation, have the potential to promote neuroplasticity and accelerate recovery from psychiatric and neurological dysfunction. Several tools are currently approved for clinical use and have been shown to induce antidepressant or reparatory effects, including transcranial magnetic stimulation (TMS), electroconvulsive therapy (ECT), and magnetic seizure therapy (MST). These methodologies induce either excitation or inhibitory on stimulated neurons as well as structurally and functionally connected circuits. Before delving into these clinical methodologies, a few basic science approaches that explicitly probe the mechanism-of-action underlying synaptic plasticity are worth considering. Single-pulse TMS protocols, for example, can be employed in conjunction with EEG or electromyogram to assess the latency and amplitude of neural responses and the mechanisms behind LTP and LTD [52]. Similarly, the paired associative stimulation protocol is an approach in which transcutaneous electrical stimulation of the median nerve is synchronously paired with single-pulse TMS over the contralateral motor cortex. In this protocol, suprathreshold stimuli are delivered to the motor [53] and prefrontal cortex [54] prior to and after repeated, paired stimuli are delivered to peripheral and cortical regions in a time window needed to generate cotemporaneous excitation of cortical output neurons. The mechanism underlying these changes is thought to be induction of Hebbian LTP-like plasticity prompted by coactivation of sensory afferents and the primary motor cortex, resulting in increased synaptic efficacy in the corticospinal motor track depending on the relative timing of the induced activations.

Another means of assessing neuroplasticity that results from clinical procedures is through the concurrent application of single-pulse TMS during recording of EEG (the so-called TMS evoked potential or TEP). TEPs can be used to characterize plasticity induced alterations after TMS treatment. For example, Hadas et al. employed TEP to investigate aberrant connectivity between the DLPFC and SGC in patients with TRD. Through analysis of significant current scattering (SCS) which measures the average distance of activated sources from the site of stimulation and significant current density (SCD) which sums of all currents induced by TMS, they were able to infer the effective connectivity and activity, respectively. Furthermore, they were able to correlate reduced SCS between the DLPFC and SGC with a reduction in depressive symptoms [55, 56]. SCS and SCD are among a few analysis methods that can be applied to the TEP to delineate physiological mechanisms in treatment induced plasticity. Going forward, use of such physiological metrics will provide added value for the interpretation of clinical outcomes that result from brain stimulation treatments and provide valuable information about circuit-level plasticity between the targeted treatment site and areas that generate the SCS and SCD.

Returning to the clinical implementations of NIBS, TMS is an effective FDA approved treatment for several psychiatric disorders including depression, obsessive-compulsive disorder, and tobacco use disorder, with other indications currently under investigation. One advantage of this therapy lies in its ability to induce plasticity long after the course of treatment. TMS uses noninvasive electromagnetic induction to stimulate neurons ~2 cm beneath the scalp. The brief local magnetic field generated by the coil creates an electric current which induces either LTP or LTD in stimulated neurons depending on the frequency [57].

One recent advance in TMS is the use of intermittent theta-burst stimulation (iTBS) which was FDA approved for MDD in 2018. This form of TMS involves bursts of gamma (50 Hz) stimulation that repeat at a theta (5 Hz) rhythm, creating a more potent treatment that can be delivered more quickly and with less burden to the patient [58]. iTBS elicits effects more efficiently as it mimics the brain’s endogenous theta rhythms, altering gamma oscillations which are necessary for neocortex-mediated cognitive functions [59], ultimately inducing long-term synaptic plasticity. Continuous TBS on the other hand, induces LTD through suppression of cortical excitability [60]. Constant, low-frequency (<1 Hz) rTMS leads to depotentiation of circuits whereas high-frequency stimulation is reported to increase neuronal firing and lead to LTP [61]. Furthermore, by combining TMS with EEG, it is possible to examine the balance between inhibition and excitation in affected brain regions which can be informative of dysregulated circuit function and guide targeted therapies. Although the TMS magnetic flux only penetrates to superficial cortical neurons, TMS can elicit its effects indirectly through connected networks [62]. Numerous clinical trials have shown that stimulating the DLPFC in depressed patients, which is frequently hypoactive in MDD, leads to its normalization of activity and subsequent improvements in mood [63, 64]. In schizophrenia, low-frequency TMS can be applied to the abnormally hyperactive temporoparietal junction to induce LTD and resolve hallucinations [65]. Additionally, functional MRI (fMRI) has revealed that dysregulated activity in deeper connected regions is also normalized through TMS for example, the subgenual cingulate cortex is hyperactive in MDD and functionally connected to the DLPFC [66]. While the mechanisms of long lasting neuromodulatory effects of NIBS remain unclear, numerous clinical trials have shown that these methods can normalize aberrant connectivity seen in psychiatric [66, 67] and addictive [68, 69] disorders.

Although the principal idea that high-frequency TMS induces LTP and low-frequencies induce LTD has been the basis of many clinical trials, the literature addressing plasticity effects in TMS is quite variable. Numerous studies have found that higher frequencies (5–20 Hz) tend to increase cortical responses [70, 71] while frequencies below 5 Hz most frequently inhibit neural responses [70, 71] and behavior however, some studies have found evidence to the contrary [72]. A systematic review conducted by Beynel et al. found that surprisingly, of the ten studies that reported results from inhibitory protocols, seven found increases in resting-state FC, one found decreased connectivity, and another found both effects [62]. On the other hand, of the 25 excitatory protocols in their review, only nine showed increased connectivity, three had decreased connectivity, and 5 did not report any changes [62]. This review points to evidence that the effects of TMS may be more diverse across networks that span different scales and across different brain areas, calling into question the simpler frequency dependent heuristic. Variations in these findings could be attributed to the connectivity between stimulated and anatomically and functionally connected regions as well as entrainment of endogenous rhythms, leading to related alterations in connectivity and neural response [62]. Therefore, when considering the effects of high-frequency versus low-frequency protocols, investigators must consider the downstream alterations on connected regions.

In addition to the non-convulsive techniques noted above, convulsive therapies induce their antidepressant effects from controlled seizures, acting through neuroplasticity. ECT is the most effective treatment for depression with a response rate of ~80% [73]. Although the therapeutic mechanism of induced seizures is not fully understood, neuroplasticity is thought to be a key player as seen through the electrically induced changes in brain volume as well as synaptogenesis, neurogenesis, and increased glial activation [74]. In MDD patients, studies have shown normalization of hippocampal and amygdala volume, within just 72 h [75]. However, contrasting results have hypothesized that ECT induced changes were due to increased hippocampal FC as opposed to brain volume, whereas others have shown no changes in FC [74]. Despite these contradictory explanations, it remains that ECT is highly effective at reducing symptoms in TRD.

MST is another convulsive therapy that has been used to alter neuroplasticity and treat depression. In this modality, a therapeutic seizure is elicited through focused magnetic fields, however, unlike ECT which produces a sequela of cognitive side effects [76], MST does not lead to as many adverse cognitive effects such as memory loss since the magnetic field only induces electrical current within the targeted brain region [77]. Although not quite as effective as ECT, studies have demonstrated improvements in clinical symptoms such as one study by Sun et al. that related reduction in suicidal ideation to MST applied to the DLPFC. They found that cortical evoked activity increased in responders, neuroplasticity was induced in the frontal cortex through LTP, and suicidal ideal scores were significantly reduced. While there are numerous studies demonstrating the efficacy of MST in treating depression, its mechanism remains to be uncovered however, neuroplasticity is thought to be key to the therapeutic benefits of the induced seizures.

While many studies have shown the advantages of NIBS in inducing LTP and LTD to resolve circuit dysfunctions, clinical results have been mixed due to inconsistent methodologies, a lack of proper controls, and multifaceted patient profiles. However, these protocols are currently being improved upon through increased individualization of stimulation parameters and combining NIBS with behavioral or pharmacological interventions to maximize plasticity.

### Reshaping synapses with cognitive behavioral therapy (CBT) and targeted cognitive training

While the previous sections have provided an overview of pharmacology and interventional approaches and their respective effects on synaptic plasticity, behavioral interventions such as CBT and targeted cognitive training have also shown the ability to modulate synaptic plasticity.

CBT is an effective, time-tested form of talk therapy based on discussion around the interplay among a patient’s thoughts, feelings and behaviors. It is highly prevalent as a treatment and used across a variety of psychiatric disorders ranging from MDD and anxiety disorders, to substance abuse and eating disorders. CBT has demonstrated changes in plasticity that are associated with clinical improvement. In one instance, 26 participants with social anxiety disorder were assigned either CBT or an attention bias modification control treatment [78]. CBT was found to yield decreases in both gray matter volume and BOLD responsivity in the amygdala, implying structural and functional level changes that decrease synaptic strength. The authors postulated that the diminished gray matter mediated the decreased responsivity and social anxiety in the CBT group, providing a specific mechanism by which plasticity may produce clinically meaningful effects in this treatment paradigm [78]. Conversely, a study of 13 patients with chronic pain who received CBT showed improved clinical measures as well as increased gray matter in the prefrontal and posterior parietal cortices as demonstrated through MRI VBM compared to 13 healthy controls [79]. These two studies taken together reinforce the premise for therapy-driven modulations of synaptic plasticity. Both studies led to clinical improvement, however through a decrease and increase in synaptic strength, respectively.

Prefrontal neural networks involved in motivation, cognition, social and emotional behavior exhibit learning-dependent plasticity. There has been considerable research on developing engaging cognitive training to induce and maximize this plasticity, yielding several key takeaways. Namely, the mechanisms behind cognitive training induced neural plasticity are present throughout life and can impact higher-order cognition, but are also impacted by behavioral states and brain chemistry [80]. From a clinical perspective, data has demonstrated large, generalizable, and durable effects, with evidence of plasticity in frontal and sensory neural networks [80].

Individuals with schizophrenia are known to have accelerated cortical thinning, and by extension synapse loss, that is associated with illness severity. A recent study examined changes in cortical thickness in patients with recent onset schizophrenia randomly allocated to either targeted cognitive training or a computer game control intervention; whereas mean cortical thickness was not significantly changed in response to targeted cognitive training, individual increases in cortical thickness were predictive of increases in global cognition—an effect not seen in the control group [81]. Previous studies in patients with schizophrenia have established that cognitive training may impact structural and functional plasticity. Notably, 40 h of auditory and working memory targeted cognitive training were associated with changes in thalamic volume in patients with schizophrenia, suggestive of either a protective or enhancing effect on thalamic structure [82]. Of these two possibilities, a model for neural response to targeted cognitive training in patients with schizophrenia is one in which the training evokes general neuroprotection, as opposed to region-specific changes in neuroplasticity [81]. This is believed to be due to the increased cortical thickness, or prevented cortical thinning, seen in patients with schizophrenia who had undergone targeted cognitive training and exhibited corresponding increased global cognitive scores [81].

The left and right middle frontal gyri (MFG) of patients with schizophrenia are known to have signaling abnormalities, exhibiting both hyper and hypoactivation when performing working memory tasks [83, 84]. Intensive cognitive training in patients with schizophrenia increased fMRI signal efficiency of MFG compared to computer game control recipients with lasting effects present at 6 months post-intervention; notably, participants had improved working memory task performance, increased activation in the left MFG, and a significant association between improved task performance and right MFG signal [85]. Once more, changes in signaling efficacy seen in the cognitive training group lends itself to the idea that neural plasticity in humans can be induced in a measurable form via deliberate training.

## Future research directions—challenges and opportunities

Understanding the roles of neuroplasticity in neuropsychiatric illnesses and their relationship to symptom development is critical to creating improved, targeted therapies. While there has been progress toward understanding the mechanisms of plasticity and innovations in therapies, there remain several challenges to studying this process. One of the major challenges is the multiscale nature in which the brain changes. Plasticity occurs on a spectrum from microscale changes at the synapse to morphological alterations that span the entire nervous system. They occur moment-to-moment and continually throughout the entire lifespan. Relating synaptic development to clinical improvement requires integration of multiple tools in conjunction, adding to the complexity of the evaluation, but creating new opportunities for discovery and development. Moreover, when comparing the brain before and after treatment, or even between healthy and diseased individuals, plasticity can depend on experience, time, and individual factors such as stress and genetics [15]. Interactions between these variables needs to be considered when attempting to delineate healthy and diseases brains. Therefore, this highly variable process complicates the generalizability of biomarkers and presents challenges to the field moving forward.

Although animal models can provide direct insight into neuronal alterations through invasive imaging and sampling, establishing valid animal models of psychiatric illnesses is difficult and means that findings remain speculative until validated in humans [86]. Animal models are also typically more homogenous in their symptomology and do not have co-morbid illnesses which tend to complicate disease etiology in humans. This is especially relevant when trying to correlate changes in plasticity to improvements in disease related behavior. On the other hand, most measures to assess plasticity in humans are noninvasive and are therefore not able to directly ascertain changes at a microscale, leaving open the possibility that they reflect indirect consequences of disordered plasticity and not the foundational pathology itself. In addition, due to the heterogenous nature of psychiatric illnesses and multiple factors influencing plasticity, establishing baseline healthy brain states, relative to disease states, is an important challenge. It is therefore challenging to assess the causes of maladaptive plasticity and distinguish these from their effects on behavior.

Despite these challenges, there is tremendous growth in the field with boundary-crossing innovations that allow greater resolution in space and time, and new approaches to link across multiple scales. In particular, there is a strong movement toward the development of predictive biomarkers of treatment efficacy that are critical for understanding not only how plasticity influences symptoms but determining who will benefit most from a given therapy. For example, by demonstrating correlations between specific markers of plasticity and individuals who respond to treatment, future clinical trials can use this information to predict whether a patient is likely to improve through changes in brain plasticity [87]. One example of a clinical biomarker currently in use is the presence of single nucleotide polymorphisms that correlate with greater efficacy of olanzapine in schizophrenia [88]. Epigenetic markers are also useful to predict drug efficacy such as the absence of methylated exon 4 of BDNF which is associated with reduced response to anti-depressants [89]. Not only would it be informative to have biomarkers of treatment response, but also of treatment progression [90]. To address some of the concerns with regards to disease heterogeneity and causes of dysregulation, it is crucial to have a better biological understanding of healthy neuroplasticity and how it can be dysregulated. Biomarkers would provide an objective measure of disease progression and pathogenesis, which could ultimately inform therapies.

Another profitable direction for future development is the individualization of treatment protocols to each patient with the goal of improving therapeutic gains. This is especially relevant to address the challenge of varied clinical profiles. For example, in most TMS protocols, patients are stimulated at the same brain region regardless of connectivity patterns or brain state during stimulation. Multiple studies, however, have shown that patients who respond best to TMS received stimulation at the node of the DLPFC most anti-correlated with the SGC [66]. Currently, there are investigations analyzing the difference between stimulating during resting-state, a more variable brain state between patients, versus during a task to create consistency or engage the network of interest [91]. Finally, combining therapies could provide another degree of personalization if patients respond better with just neurostimulation or with behavioral or pharmacological interventions as well. Biomarker development and improved therapeutic tools have the potential to transform patient outcomes through treatment personalization and objective measure of neuroplasticity.

Collectively, there is a profound need consistency in methodologies of interventions for psychiatric disorders. Currently response and remission rates remain low which may be due in part to the lack of proper controls, highly variable treatment parameters, and lack of consistency among clinical symptoms of patients [92]. By combining functional and structural imaging tools, therapies can be tailored to the individual. These and other innovations described in the preceding sections will continue to drive the field forward and to create more effective and efficient treatments
